# Nicorandil attenuates thioacetamide induced liver fibrosis via AMPK, SIRT1 and HIF1α mediated cellular energy homeostasis

**DOI:** 10.1038/s41598-025-28309-7

**Published:** 2025-12-09

**Authors:** Magy R. Kozman, Mohamed Gamal El-Di Ewees, Yasmin M. Ahmed, Ehab A. M. El-Shoura, Saad Misfer Alqahtani, Lamiaa Khalaf Ahmed, Omar A. Farghaly, Mostafa Sabry, Yasmine H. Ahmed, Fatma El-Zahraa S. Abdel Rahman, Ahmed M. Atwa

**Affiliations:** 1 Clinical Pharmacy Department, Faculty of Pharmacy, Misr University for Science and Technology, Giza, 12563 Egypt; 2 Department of Pharmacology and Toxicology, College of Pharmacy, Almaaqal University, Basrah, 61014 Iraq; 3 Department of Pharmacology, Faculty of Pharmacy, Nile Valley University Egypt, Fayoum, 63518 Egypt; 4 Department of Clinical Pharmacy, Faculty of Pharmacy, Assiut Branch, Al-Azhar University, Assiut, 71524 Egypt; 5 Department of Pathology, College of Medicine, The University Hospital, Najran University, Najran, Saudi Arabia; 6 Department of biochemistry and Molecular Biology, Faculty of Pharmacy (Girls), Al-Azhar University, Cairo, Egypt; 7 Department of Biochemistry and Molecular Biology, Faculty of Pharmacy, Assiut Branch, Al-Azhar University, Cairo, Egypt; 8 Department of Cytology and Histology, Faculty of Veterinary Medicine, Cairo University, Giza, Egypt; 9 Department of Basic Sciences, Faculty of Oral and Dental Medicine, Nahda University, Beni-Suef, 62764 Egypt; 10 Pharmacology and Toxicology Department, Faculty of Pharmacy, Egyptian Russian University, Cairo, 11829, Egypt

**Keywords:** Nicorandil, Thioacetamide, Fibrosis, AMPK, SIRT-1, HIF-1α, Diseases, Drug discovery, Gastroenterology, Medical research

## Abstract

Liver fibrosis, marked by excessive extracellular matrix deposition and chronic inflammation, is a significant health concern that can progress to cirrhosis and liver failure. Current treatments are limited, highlighting the need for novel therapies. Nicorandil (NIC), a potassium channel opener with nitric oxide-donating properties, has shown cytoprotective and anti-inflammatory effects. This study evaluates the efficacy of NIC in attenuating thioacetamide (TAA)-induced liver fibrosis in rats, focusing on the AMPK/SIRT-1/HIF-1α signaling pathway. Twenty-four adult male albino rats were randomly assigned to four groups: a control group, a TAA-treated group, and two groups treated with NIC at doses of 7.5 mg/kg/day and 15 mg/kg/day, respectively, for six weeks. TAA was administered intraperitoneally for six weeks to induce fibrosis, while NIC was given concurrently at both doses. TAA administration caused marked liver injury and fibrosis, as shown by increased ALT, AST, collagen-1α, and hydroxyproline levels. NIC treatment, particularly at 15 mg, significantly reduced these markers, indicating improved liver function and less fibrosis. NIC also alleviated oxidative stress by increasing SOD and GSH while lowering MDA, NO₂⁻, and iNOS. At the molecular level, NIC upregulated AMPK, SIRT-1, P53, and PGC-1α, and downregulated HIF-1α and STAT3. It further suppressed pro-inflammatory cytokines (IL-6, IL-1β, TNF-α, NF-κB, TGF-β1) while elevating IL-10. Histopathology confirmed improved liver structure with reduced collagen deposition, and immunohistochemistry showed decreased COX-II expression. In conclusion, both NIC doses showed therapeutic potential, with the 15 mg dose demonstrating superior efficacy. These findings highlight NIC’s promise as an effective treatment for liver fibrosis by mediating the AMPK/SIRT-1/HIF-1α pathway and modulating key molecular and cellular processes.

**Supplementary Information:**

The online version contains supplementary material available at 10.1038/s41598-025-28309-7.

## Introduction

Liver fibrosis is a progressive pathological condition that poses a significant global health challenge, arising from various chronic liver insults such as viral infections, metabolic disorders, alcohol consumption, and toxic exposure^1^. It is a progressive condition characterized by excessive extracellular matrix (ECM) deposition, which disrupts the normal architecture of the liver and impairs its function. If left untreated, liver fibrosis can progress to cirrhosis, a terminal stage of liver disease that leads to liver failure, portal hypertension, and an increased risk of hepatocellular carcinoma^2^.

Despite its prevalence, there is still a lack of effective antifibrotic therapies, with current treatments mainly targeting the underlying causes of liver injury rather than addressing the fibrotic process itself^3^. Thus, the need for new therapeutic strategies to halt or even reverse fibrosis progression is pressing. One promising avenue involves liver regeneration, a complex and coordinated process by which hepatocytes and other liver cells proliferate to restore liver function after injury. This regenerative capacity, when appropriately activated, may offer a mechanism to reverse fibrosis, especially in the early stages of liver injury^4^. Several signaling pathways, such as those regulating inflammation, metabolism, and cell proliferation, play key roles in liver injury and regeneration, providing potential targets for antifibrotic therapies aimed at stimulating liver repair while inhibiting fibrogenesis^5^.

One widely used experimental model of liver fibrosis is the thioacetamide (TAA)-induced fibrosis model in rodents, which closely mimics the histopathological features of human liver fibrosis^6^. Chronic administration of TAA leads to oxidative stress, hepatocyte necrosis, and inflammation, ultimately activating hepatic stellate cells (HSCs), the primary effector cells in fibrosis. Once activated, HSCs undergo trans differentiation into myofibroblasts, which secrete collagen and other ECM components, driving the progression of fibrosis^7^. In addition to the activation of HSCs, TAA exposure promotes inflammation, as evidenced by the release of pro-inflammatory cytokines such as Tumor necrosis factor-alpha (TNF-α) and Interleukin-6 (IL-6), which further exacerbate liver damage and promote fibrosis^7^. Furthermore, the imbalance between reactive oxygen species (ROS) and the liver’s antioxidant defense mechanisms contributes to the pathogenesis of fibrosis, amplifying both hepatocellular injury and the fibrotic response^8^. This model, therefore, serves as an excellent tool to study the molecular mechanisms underlying liver fibrosis and to test potential therapeutic interventions.

Recent research has increasingly focused on understanding the molecular pathways involved in both liver fibrosis and regeneration. One of the central regulators of cellular energy metabolism is AMP-activated protein kinase (AMPK), which plays a key role in regulating metabolic homeostasis^9^. AMPK functions as a metabolic sensor that responds to changes in cellular energy levels by activating catabolic pathways and inhibiting anabolic processes. In the context of liver fibrosis, AMPK activation has been shown to have protective effects, as it not only reduces HSC activation and ECM production but also promotes liver regeneration by stimulating hepatocyte proliferation and mitochondrial function^10^. Furthermore, AMPK activation can help balance the fibrotic and regenerative processes within the liver, making it a potential therapeutic target for reversing liver fibrosis^11^.

Sirtuin 1 (SIRT-1), a NAD+-dependent deacetylase, plays a complementary role in this process. SIRT-1 is known to regulate several transcription factors involved in inflammation and fibrosis, including Nuclear factor kappa-light-chain-enhancer of activated B cells (NF-κB) and Peroxisome proliferator-activated receptor gamma (PPAR-γ), thus modulating the fibrotic response^12^. Additionally, SIRT-1 interacts with AMPK, with evidence suggesting that their coordinated activity synergistically reduces fibrosis and enhances tissue regeneration. The interaction between AMPK and SIRT-1, therefore, represents an important axis for managing liver fibrosis and stimulating repair^13^. Moreover, hypoxia-inducible factor 1-alpha (HIF-1α), a key regulator of cellular responses to low oxygen conditions, also influences liver fibrosis, especially under hypoxic stress^14^. Under such conditions, HIF-1α activates genes involved in fibrosis and ECM deposition. However, recent studies suggest that AMPK and SIRT-1 can modulate HIF-1α activity, promoting a balance between fibrosis and regeneration, which is crucial for effective liver repair^15^.

One such potential therapeutic agent is NIC, a potassium channel opener and nitric oxide (NO) donor that has primarily been used in the management of cardiovascular diseases. However, emerging evidence suggests that NIC may also have beneficial effects in liver diseases, particularly in fibrosis^16,17^. By opening ATP-sensitive potassium (K_ATP) channels and donating NO, NIC improves blood flow and reduces ischemic damage, which could help mitigate the effects of liver fibrosis. Furthermore, NIC has been shown to modulate the AMPK/SIRT-1 /HIF-1α pathway, potentially exerting anti-fibrotic and pro-regenerative effects^18^. Despite these promising findings, the role of NIC in liver fibrosis remains largely unexplored, particularly in the context of TAA-induced liver injury. Therefore, the current study aims to investigate the therapeutic potential of NIC in TAA-induced liver fibrosis in rats, focusing on its ability to regulate the AMPK/SIRT-1/HIF-1α axis and promote liver regeneration. This study seeks to fill a critical gap in existing literature and provide new insights into the molecular mechanisms by which NIC may offer a novel strategy for treating liver fibrosis.

## Materials and methods

### Drugs, chemicals, and kits

Thioacetamide (TAA) was purchased from Sigma-Aldrich (St. Louis, MO, USA), and Nicorandil (NIC) was kindly provided by Adwia Co. (Cairo, Egypt). For daily administration, both compounds were dissolved in sterile normal saline containing 1% Tween 80. Commercially available diagnostic kits for the quantitative assessment of hepatic enzyme activities—namely aspartate aminotransferase (AST; Cat. No: AS 10 61) and alanine aminotransferase (ALT; Cat. No: AL 10 31)—were obtained from Bio-Diagnostic Co. (Giza, Egypt). Furthermore, colorimetric assay kits for evaluating oxidative and nitrosative stress biomarkers in liver tissue were sourced from the same supplier. These included kits for the determination of nitric oxide metabolites (NOx; Cat. No: NO 25 33), malondialdehyde (MDA; Cat. No: MD 25 29), reduced glutathione (GSH; Cat. No: GR 25 11), and superoxide dismutase (SOD; Cat. No: SD 25 21).

ELISA kits specific for rat pro- and anti-inflammatory cytokines were acquired from ELK Biotechnology Co., Ltd. (Wuhan, China). These included kits for interleukin-6 (IL-6; Cat. No: ELK1158), interleukin-10 (IL-10; Cat. No: ELK1144), interleukin-1β (IL-1β; Cat. No: ELK1272), tumor necrosis factor-alpha (TNF-α; Cat. No: ELK1396), transforming growth factor-beta 1 (TGF-β1; Cat. No: ELK2311), inducible nitric oxide synthase (iNOS; Cat. No: ELK1503), and hydroxyproline (HYP; Cat. No: ELK8687). Additionally, the bicinchoninic acid (BCA) protein assay kit (Cat. No. 23225, Thermo Fisher Scientific, USA) for the western blotting assay. All other chemicals and reagents used throughout the study were of analytical grade and sourced from standard commercial suppliers.

### Animals

A total of twenty-four adult male albino rats, weighing between 150 and 170 g, were obtained from the Animal house facility of the Faculty of Medicine, Assiut University (Assiut, Egypt). Prior to experimentation, animals were housed under standard laboratory conditions for a one-week acclimatization period. Environmental conditions were maintained at a controlled temperature of 22 ± 3 °C, relative humidity of 55 ± 5%, and a 12-hour light/dark photoperiod. Throughout the study, rats had *ad libitum* access to a standard pellet diet and fresh water. To keep the rats healthy during the study, we used straw pellet bedding that was changed daily.

All animal handling procedures and experimental protocols conformed to the international guidelines for the ethical use and care of laboratory animals, as outlined in the European Directive 2010/63/EU and the earlier Council Directive 86/609/EEC. The study protocol was reviewed and approved by the Research Ethics Committee of the Faculty of Pharmacy, Al-Azhar University, Assuit Branch, in accordance with national regulations. The ethical approval reference number is (ZA-AS/PH/5/C/2023).

### Experimental design and sample collection

Animals were randomly allocated into four experimental groups (*n* = 6 per group) as follows:

Group I (Normal control): Received intraperitoneal (i.p.) injections of sterile normal saline twice weekly for six consecutive weeks.

Group II (TAA): Administered TAA at a dose of 250 mg/kg, *i.p*., twice weekly for six weeks to induce hepatic fibrosis, as previously described^19,20^.

Group III (TAA + NIC 7.5 mg): Received TAA as in Group II, in addition to NIC orally at a dose of 7.5 mg/kg/day, concurrently for six weeks^21^.

Group IV (TAA + NIC 15 mg): Treated similarly to Group III, but with a higher oral dose of NIC (15 mg/kg/day), administered throughout the six-week experimental period^22^.

At the end of the treatment period, blood samples were collected from the retro-orbital plexus under anesthesia through an intraperitoneal injection of a ketamine (50 mg/kg) and xylazine (10 mg/kg) mixture. Samples were immediately centrifuged at 4000 rpm for 20 min, and the separated sera were preserved at − 20 °C for subsequent biochemical assays, including ALT and AST. Subsequently, rats were euthanized by carbon dioxide inhalation. Liver tissues were promptly excised, rinsed in ice-cold normal saline to remove excess blood, and sectioned into two portions. One portion was snap-frozen in liquid nitrogen and stored at − 80 °C for biochemical and molecular analyses. The remaining portion was fixed in 10% neutral buffered formalin for histopathological examination and immunohistochemical staining. Investigators responsible for histopathological assessment, immunohistochemistry, and biochemical analyses were blinded to the treatment assignments during data collection and statistical analysis.

### Estimation of liver function tests

Serum activities of ALT and AST were quantitatively measured using commercially available colorimetric assay kits, following the manufacturer’s protocols (Bio-Diagnostic, Giza, Egypt), following the method of Retman and Frankel 1957. Briefly, 1 ml of the rat serum was incubated in a water bath for 10 min at 40**°**C. Then, 0.2 mL of serum was added to the reagent mixture, mixed thoroughly, and incubated for 60 min for AST or 30 min for ALT. After incubation, 1 mL of 2,4-dinitrophenylhydrazine (DNPH) reagent was added immediately, and the tubes were kept at room temperature for 20 min. Subsequently, 10 mL of 0.4 N sodium hydroxide (NaOH) was added, mixed gently, and allowed to stand for 30 min. The optical density (OD) was measured at 505 nm using a spectrophotometer. Enzyme activities were calculated and expressed as U/L serum activity.

### Estimation of reactive oxidative stress parameters

Liver tissue homogenates were prepared and subjected to biochemical analysis to quantify lipid peroxidation and antioxidant defense parameters. The levels of MDA, GSH, and NOx, along with SOD activity, were determined using specific colorimetric spectrophotometric methods. Lipid peroxidation was evaluated by quantifying MDA levels. In brief, 5 µL of each sample was mixed with 45 µL of 50 mM monobasic sodium phosphate buffer in 1.5 mL microtubes. Subsequently, 12.5 µL of 8.1% SDS, 93.5 µL of 20% trichloroacetic acid (pH 3.5), and 93.5 µL of 1% thiobarbituric acid were added, followed by 50.5 µL of ultrapure water. The mixtures were vortexed for 30 s and incubated in boiling water for 10 min, then cooled on ice. After cooling, 62.5 µL of ultrapure water and 312.5 µL of n-butanol–pyridine (15:1, v/v) were added, and the tubes were centrifuged at 5000×g for 5 min. Aliquots (150 µL) of the resulting supernatant were transferred in duplicate to a 96-well microplate, and absorbance was measured at 520–530 nm. A standard calibration curve was constructed using bis(dimethyl acetal) MDA, and results were expressed as nmol MDA per gram of tissue protein.

### Estimation of inflammatory and Proinflammatory cytokines

Pro-inflammatory and anti-inflammatory cytokines, including IL-6, IL-10, IL-1β, TNF-α, TGF-β1, iNOS, and HYP, were measured in liver tissue samples using rat-specific enzyme-linked immunosorbent assay (ELISA) kits obtained from ELK Biotechnology (Wuhan, China) following to manufacture instructions through using Microplate reader equipment (India). All assays were performed in duplicate to ensure data reliability. Absorbance was measured at the appropriate wavelength using a microplate reader (ELx800™, BioTek Instruments, USA).

### Hepatic mRNA gene expression

Total RNA was isolated from liver tissue using standard extraction protocols, followed by reverse transcription to synthesize complementary DNA (cDNA), as previously described^23^. Quantitative real-time polymerase chain reaction (qRT-PCR) was performed using the 7300 Real-Time PCR System (Applied Biosystems, Foster City, CA, USA) to assess the relative mRNA expression levels of nuclear factor kappa B p65 (NF-κB p65), tumor suppressor protein p53 (P53), and collagen type I alpha 1 (COL1α1). β-actin was used as the endogenous reference gene to normalize target gene expression. Primer sequences employed in the reactions are listed in Table 1. Relative gene expression was calculated using the 2^–ΔΔCt method. Primer specificity was confirmed by melting-curve analysis and agarose gel electrophoresis, which revealed single amplicons of the expected size. Amplification efficiency for each primer pair was determined by standard curve analysis using serial cDNA dilutions. All primers exhibited efficiencies within 90–110% and correlation coefficients (R^2^) > 0.99.

**Table 1:** The primer sets for targeted genes.

Gene	Primers
NF-ҡB-p65	5′- TGCAGGCTCCTGTGCGAGTG -3
	5′- TCCGGTGGCGATCGTCTGTGT -3
Collagn-1α	5′ ATGTTCAGCTTTGTGGACCT-3′
	5′-CAGCTGACTTCAGGGATGT-3′
GAPDH	5′- AGGGAAATCGTGCGTGACAT -3′

### Western blotting Estimation

Total protein concentrations from liver homogenates were determined using the bicinchoninic acid (BCA) protein assay kit (Cat. No. 23225, Thermo Fisher Scientific, USA). Equal amounts of protein were separated on SDS-polyacrylamide gels (7–12%, depending on molecular weight) and electrotransferred onto polyvinylidene difluoride (PVDF) membranes (Bio-Rad, Hercules, CA, USA). Membranes were blocked with 5% non-fat dry milk in TBST buffer and incubated overnight at 4 °C with primary antibodies against AMPK, SIRT-1, HIF-1α, PGC-1α, PPARγ, STAT3 (each at 1:1000 dilution), and β-actin as the loading control. Antibodies were obtained from Santa Cruz Biotechnology, Chongqing Biospes Co., Ltd, Abbexa Ltd, and Elabscience. After incubation with horseradish peroxidase-conjugated secondary antibodies, protein bands were visualized using an enhanced chemiluminescence (ECL) detection system (Tanon-5200Multi, Shanghai, China). Band intensity was quantified using ImageJ software (version 1.8.0, NIH, USA). Western blot analyses were performed using liver tissue homogenates from six rats per group, and each experiment was conducted in triplicate (three independent repeats) to ensure reproducibility. Representative blots are shown in the figures.

### Histopathological and Masson trichrome stain examination

Liver specimens fixed in 10% neutral buffered formalin were processed, embedded in paraffin wax, and sectioned at 4 μm thickness. Sections were stained with hematoxylin and eosin (H&E) for general histological examination and with Masson’s trichrome stain to evaluate collagen deposition and fibrosis (Bancroft and Gamble, 2013). Microscopic evaluation was performed using an Olympus BX50 light microscope (Olympus, Japan), and digital images were captured with an Olympus imaging system at a magnification of 400×. Masson Trichrome stained hepatic sections were analyzed using image analysis software (LEICA microsystems (LAS version 3.8.0 (build:878), Leica Ltd) image analyzer computer system) at Cytology and Histology Department, Faculty of Veterinary Medicine, Cairo University. Histochemical staining was presented as a percent of the total area in a standard measuring frame over ten independent fields from different slides in each group at 400x magnification. All areas with positive histochemical staining reactions were chosen for evaluation.

### Immunohistochemical assay

Immunohistochemical detection of cyclooxygenase-2 (COX-2) expression in liver tissues was performed according to the method described by Merz et al. (1995). Briefly, paraffin-embedded sections were deparaffinized, rehydrated, and incubated with anti-COX-2 primary antibodies, followed by secondary antibody incubation. Immunoreactivity was visualized using a diaminobenzidine (DAB)/hydrogen peroxide substrate, and counterstaining was performed with hematoxylin. Slides were examined under a light microscope in consultation with a qualified histopathologist^24^. Anti-COX2 immunohistochemical sections were used to measure the mean area percent in different slides (*n* = 5 fields/group) using image analysis software (LEICA microsystems (LAS version 3.8.0 (build:878), Leica Ltd) image analyzer computer system) at Cytology and Histology Department, Faculty of Veterinary Medicine, Cairo University.

### Statistical analysis

Data were statistically analyzed using SPSS software, version 22.0 (IBM Corp., Armonk, NY, USA). Results were expressed as mean ± standard error of the mean (SEM). Group comparisons were conducted using one-way analysis of variance (ANOVA), followed by the least significant difference (LSD) post hoc test for multiple comparisons. A p-value less than 0.05 was considered statistically significant.

## Results

### Effect of Nicorandil on liver function tests; ALT and AST in rats with thioacetamide-induced liver fibrosis

Our results showed that levels of ALT and AST increased approximately 4.3-fold and 3.4-fold, respectively, in the TAA-induced fibrosis group compared to the normal control group, indicating substantial liver damage (Table 2). Meanwhile, treatment of animals with NIC at 7.5 mg/kg reduced ALT levels by about 39% and AST levels by approximately 34% compared to the TAA-induced fibrosis group. Additionally, a higher dose of NIC (15 mg/kg) further decreased serum ALT and AST levels, achieving reductions of around 51% and 48%, respectively, relative to the TAA-induced fibrosis group. This dose also showed a significant improvement compared to the 7.5 mg dose, indicating a dose-dependent protective effect of NIC on liver function in TAA-induced liver fibrosis (Table 2).

**Table 2:** Effect of Nicorandil on liver function tests; ALT and AST in rats with thioacetamide-induced liver fibrosis.

Groups	Serum ALT (U/L)	Serum AST (U/L)
Normal control	30.49 ± 1.74	34.35 ± 1.21
TAA	130.2 ± 3.45^a^	116 ± 1.39^a^
TAA + NIC 7.5 mg	79.66 ± 1.86^ab^	77.01 ± 1.63^ab^
TAA + NIC 15 mg	63.31 ± 1.89^abc^	60.26 ± 2.77^abc^

Each value represents the mean of 6-8 experiments ± SE.

*NIC* Nicorandil, *TAA* Thioacetamide.

Statistical analysis was performed using one-way ANOVA followed by Tukey-Kramer multiple comparisons test where:

^a^Significantly different from Normal control group at *p* < 0.05.

^b^Significantly different from TAA-induced fibrosis group at *p* < 0.05.

^c^Significantly different from TAA+NIC 7.5 mg group at *p* < 0.05.

### Effect of nicorandil on oxidative stress biomarkers in rats with thioacetamide-induced liver fibrosis

In this study, we observed that induction of liver fibrosis with TAA resulted in significant oxidative stress, as reflected by profound alterations in antioxidant and oxidative damage biomarkers. GSH content and SOD activity in the TAA-induced fibrosis group were markedly diminished, showing reductions of approximately 80.6% and 82%, respectively, compared to the normal control group. These decreases highlight a depletion of the liver’s antioxidant defenses under fibrotic conditions. In contrast, oxidative damage markers, MDA content and NOx activity, exhibited sharp increases, with MDA levels rising nearly 6.8-fold and NOx levels by approximately 3.8-fold compared to controls, underscoring the oxidative burden associated with TAA-induced fibrosis (Table 3).

**Table 3:** Effect of Nicorandil on oxidative stress biomarkers in rats with thioacetamide-induced liver fibrosis.

Groups	GSH (μmol/g tissue)	SOD (U/g tissue)	MDA (nmol/g tissue)	NOx (μmol/g tissue)
Normal control	37.99 ± 1.56	35.62 ± 1.55	6.27 ± 0.60	8.18 ± 0.47
TAA	7.30 ± 0.56^a^	6.40 ± 0.63^a^	42.56 ± 2.81 ^a^	31.01 ± 1.13 ^a^
TAA + NIC 7.5 mg	17.77 ± 0.83^ab^	16.42 ± 1.15^ab^	22.77 ± 1.66 ^ab^	17.49 ± 0.66 ^ab^
TAA + NIC 15 mg	26.24 ± 1.64^abc^	27.38 ± 1.03^abc^	12.22 ± 0.87 ^bc^	11.03 ± 0.93 ^bc^

Each value represents the mean of 6-8 experiments ± SE.

*NIC* nicorandil, *TAA* thioacetamide.

Statistical analysis was performed using one-way ANOVA followed by Tukey-Kramer multiple comparisons test where:

^a^Significantly different from Normal control group at *p* < 0.05.

^b^Significantly different from TAA-induced fibrosis group at *p* < 0.05.

^c^Significantly different from TAA+NIC 7.5 mg group at *p* < 0.05.

On the other hand, NIC treatment at 7.5 mg partially restored antioxidant levels, with GSH and SOD increasing by 143% and 156%, respectively, and reduced oxidative damage markers, with MDA and NOx decreasing by 46% and 44% relative to TAA-induced liver fibrosis group. The 15 mg dose further improved these markers, increasing GSH by 259% and SOD by 334% and reducing MDA and NOx by 71% and 64%, respectively. These findings suggest that NIC provides dose-dependent protection against oxidative stress in TAA-induced liver fibrosis (Table 3).

### Effect of nicorandil on the expression of TGF-1β, and HYP protein, as well as COL1α1 in hepatic tissue of rats with thioacetamide-induced liver fibrosis

**Fig. 1:**
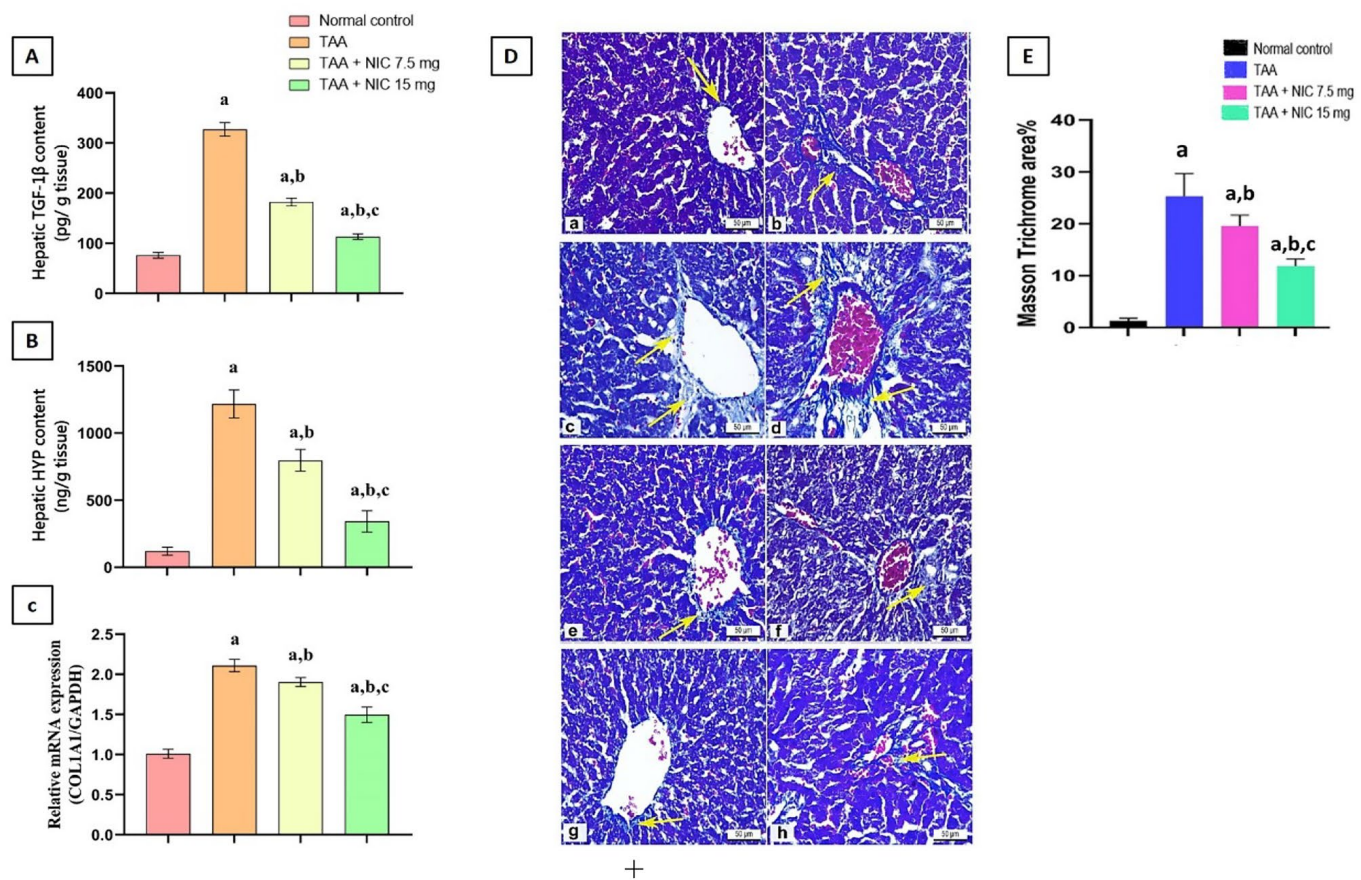
Effect of nicorandil (NIC) on the expression of TGF-β1 and hydroxyproline (HYP) proteins, gene expression of COL1α1, and collagen deposition in hepatic tissue of rats with thioacetamide (TAA)-induced liver fibrosis. **(A)** TGF-β1 protein content, **(B)** HYP content, **(C)** Relative mRNA expression of COL1α1, **(D)** Representative photomicrographs of liver tissue sections stained with Masson’s trichrome (magnification ×400). Collagen fibers are stained blue. (**a**,** b**) Normal control group showing minimal collagen deposition around the central vein. (**c**,** d**) TAA-treated group exhibiting extensive collagen deposition (blue staining) throughout the hepatic parenchyma, particularly around the central veins and within the sinusoidal spaces (indicated by yellow arrows). (**e**,** f**) TAA + NIC 7.5 mg/kg treated group showing a noticeable reduction in collagen deposition compared to the TAA group. (**g**,** h**) TAA + NIC 15 mg/kg treated group demonstrating a further marked decrease in collagen deposition, approaching the normal control levels, and **(E)** Quantitative analysis of the Masson’s trichrome-positive area (%) in different experimental groups, reflecting the extent of collagen deposition. Data are presented as mean ± standard error of the mean (SEM). Statistical significance was determined by one-way analysis of variance (ANOVA) followed by the appropriate post-hoc test.

Our results showed that TAA administration significantly elevated hepatic fibrosis markers, with TGF-β1 content rising by approximately 4.3-fold, HYP content by roughly 4.5-fold, and COL1α1 mRNA expression by 2.6-fold compared to the normal control group, indicating substantial fibrotic damage (Fig. 1A–C).

On the other hand, NIC treatment at a dose of 7.5 mg/kg markedly reduced these fibrosis markers; TGF-β1 levels, HYP content, and COL1α1 expression by 54.75%, 46.52%, and by 35.09%, respectively, relative to the TAA-induced liver fibrosis group. Moreover, the higher dose of NIC (15 mg/kg) further improved these parameters, reducing TGF-β1 levels by 70.45%, HYP content by 67.04%, and COL1α1 expression by 50.03% compared to the TAA–induced liver fibrosis group (Fig. 1A–C). These reductions correspond to a return toward normal levels, suggesting a dose-dependent anti-fibrotic effect of NIC, with the 15 mg/kg dose.

### Effect of nicorandil on the collagen deposition in hepatic tissue of rats with thioacetamide-induced liver fibrosis

Our results showed that TAA administration markedly increased collagen deposition in hepatic tissue, as evidenced by the Masson’s trichrome staining (MTC) (Fig. 1D) and quantified in the MTC-positive area (Fig. 1E). In the TAA-induced liver fibrosis group, collagen deposition was significantly elevated specially around both the central vein and portal area, with MTC area increasing by approximately 35-fold compared to the normal control, indicating extensive fibrosis.

Meanwhile, rats treated with NIC showed significant reduction in collagen deposition in hepatic tissue in a dose-dependent manner. The 7.5 mg/kg dose of NIC reduced the MTC-positive area by 45.71% relative to the TAA-induced liver fibrosis group, while the 15 mg/kg dose provided an even greater effect, decreasing collagen deposition by 65.71% comparing to the TAA-induced liver fibrosis group (Fig. 1D,E).

### Effect of nicorandil on the expression of SIRT-1, AMPK, PGC1-α, HIF-1, STAT 3, and PPARγ proteins in hepatic tissue of rats with thioacetamide-induced liver fibrosis

**Fig. 2:**
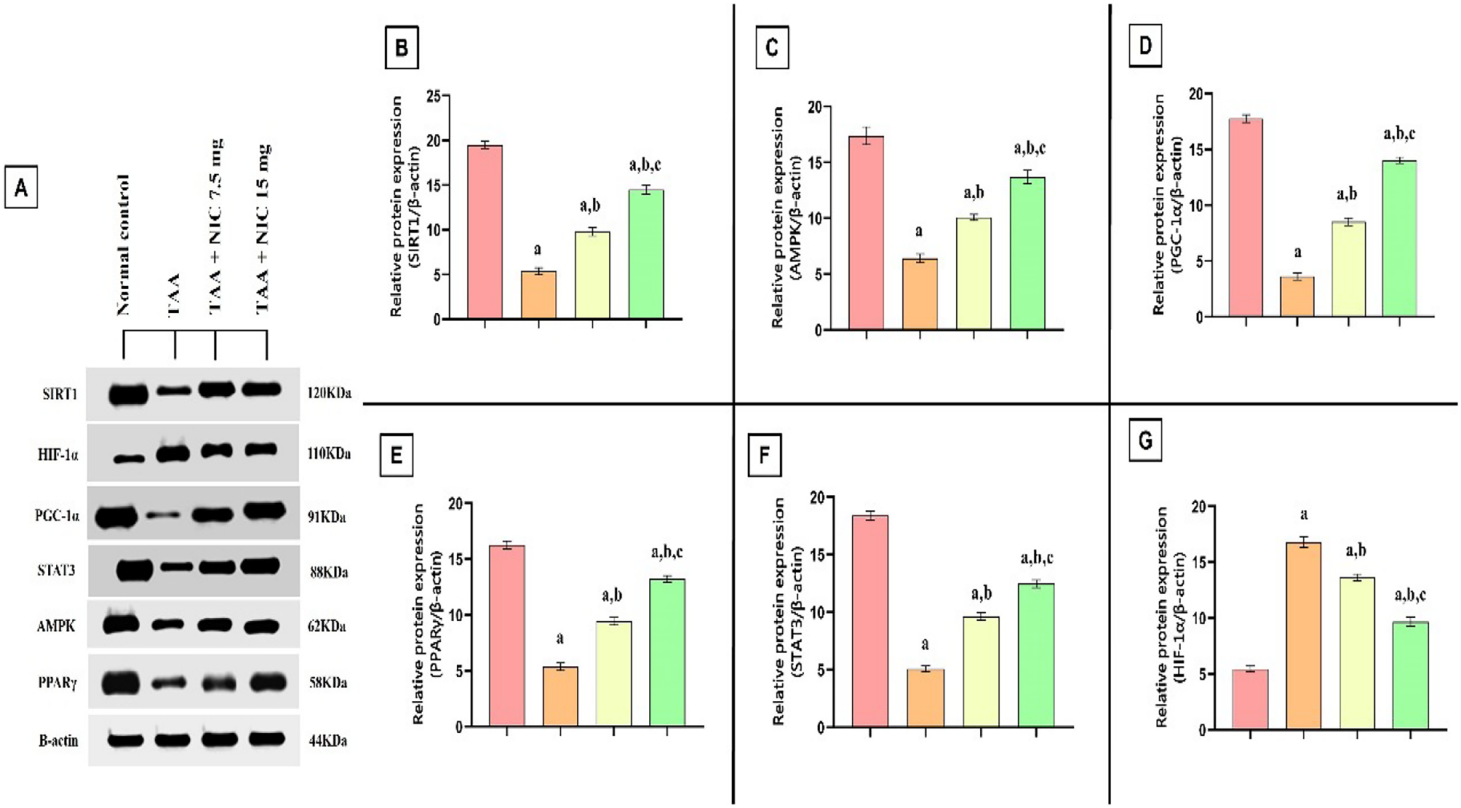
Effect of nicorandil (NIC) on hepatic protein expression of SIRT-1, AMPK, PGC-1α, STAT3, PPARγ, and HIF-1α in thioacetamide (TAA)-induced liver fibrosis in rats. **(A)** Representative Western blot images showing the hepatic expression levels of SIRT-1, HIF-1α, PGC-1α, STAT3, AMPK, and PPARγ. β-actin was used as a loading control. **(B–G)** Bar graphs represent the densitometric quantification of the relative protein expression of SIRT-1 **(B)**, AMPK **(C)**, PGC-1α **(D)**, PPARγ **(E)**, STAT3 **(F)**, and HIF-1α **(G)**, normalized to β-actin. Data are expressed as mean ± SEM (*n* = 6 per group). ^a^Significantly different from the normal control group (*p* < 0.05). ^b^Significantly different from the TAA group (*p* < 0.05). ^c^Significantly different from the TAA + NIC (7.5 mg/kg) group (*p* < 0.05).

Our findings reveal that TAA exposure caused significant disruption in hepatic protein homeostasis, resulting in substantial decreases in the expression of key proteins involved in metabolic regulation and inflammation. Specifically, the levels of SIRT-1, AMPK, PGC1-α, PPARγ, and STAT3 were reduced by 76.01%, 78.00%, 82.00%, 67.03%, and 72.37%, respectively, compared to the normal control group (Fig. 2A–F). In contrast, TAA exposure resulted in a marked 3.18-fold increase in HIF-1α expression, highlighting a heightened fibrogenic and hypoxic stress response (Fig. 2G).

However, NIC administration reversed these alterations in a dose-dependent manner. At the 7.5 mg/kg dose, NIC significantly enhanced the expression of SIRT-1, AMPK, PGC1-α, PPARγ, and STAT3 by 2.43-, 2.56-, 3.14-, 1.76-, and 1.90-fold, respectively, compared to the TAA-induced liver fibrosis group, and reduced HIF-1α expression by 28.23%. Notably, the higher NIC dose further amplified the recovery, increasing the expression of these proteins by 3.52-, 3.85-, 4.48-, 2.47-, and 2.46-fold, while HIF-1α expression was reduced by 57.89% compared to the TAA-induced liver fibrosis group (Fig. 2A,G).

### Effect of nicorandil on the expression of cytokines; IL-6, IL-1B, and IL-10 in hepatic tissue of rats with thioacetamide-induced liver fibrosis

**Fig. 3:**
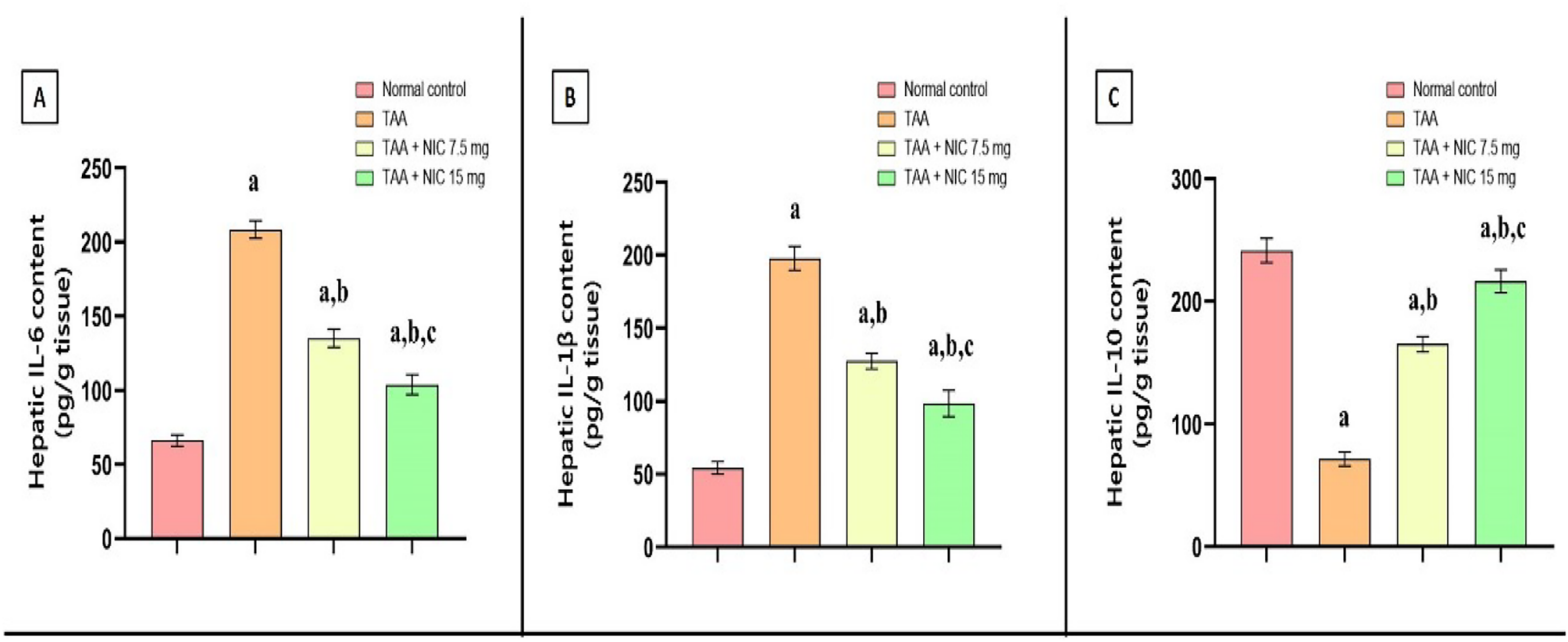
Effect of nicorandil (NIC) on hepatic levels of pro- and anti-inflammatory cytokines (IL-6, IL-1β, and IL-10) in rats with thioacetamide (TAA)-induced liver fibrosis. Bar graphs represent the hepatic content of **(A)** IL-6, **(B)** IL-1β, and **(C)** IL-10, expressed as pg/g of liver tissue. Data are expressed as mean ± SEM (*n* = 6 per group). ^a^Significantly different from the normal control group (*p* < 0.05). ^b^Significantly different from the TAA group (*p* < 0.05). ^**c)**^ Significantly different from the TAA + NIC (7.5 mg/kg) group (*p* < 0.05).

In this study, we observed that TAA administration significantly altered cytokine profiles in hepatic tissue, as evidenced by a 3.60-fold increase in IL-6 levels and a 3.75-fold increase in IL-1β levels compared to the normal control group (Fig. 3A,B). Conversely, administration of TAA significantly reduced IL-10 levels by 68.00% as compared to normal control group (Fig. 3C).

Meanwhile, treatment of rats with NIC demonstrated a dose-dependent ameliorative effect on these alterations. As it is shown in Fig. 3, The 7.5 mg/kg dose of NIC reduced IL-6 and IL-1β levels by 40.77% and 35.29%, respectively, while increasing IL-10 levels by 163.64%, relative to the TAA-induced liver fibrosis group. Similarly, the 15 mg/kg dose exhibited greater efficacy, reducing IL-6 and IL-1β levels by 54.62% and 47.06%, respectively, and enhancing IL-10 levels by 227.27% as compared to TAA-induced liver fibrosis group (Fig. 3A–C).

### Effect of nicorandil on the protein expression of TNF-α and iNOS as well as the gene expression of NF-κB-p65 and P53 in hepatic tissue of rats with thioacetamide-induced liver fibrosis

**Fig. 4:**
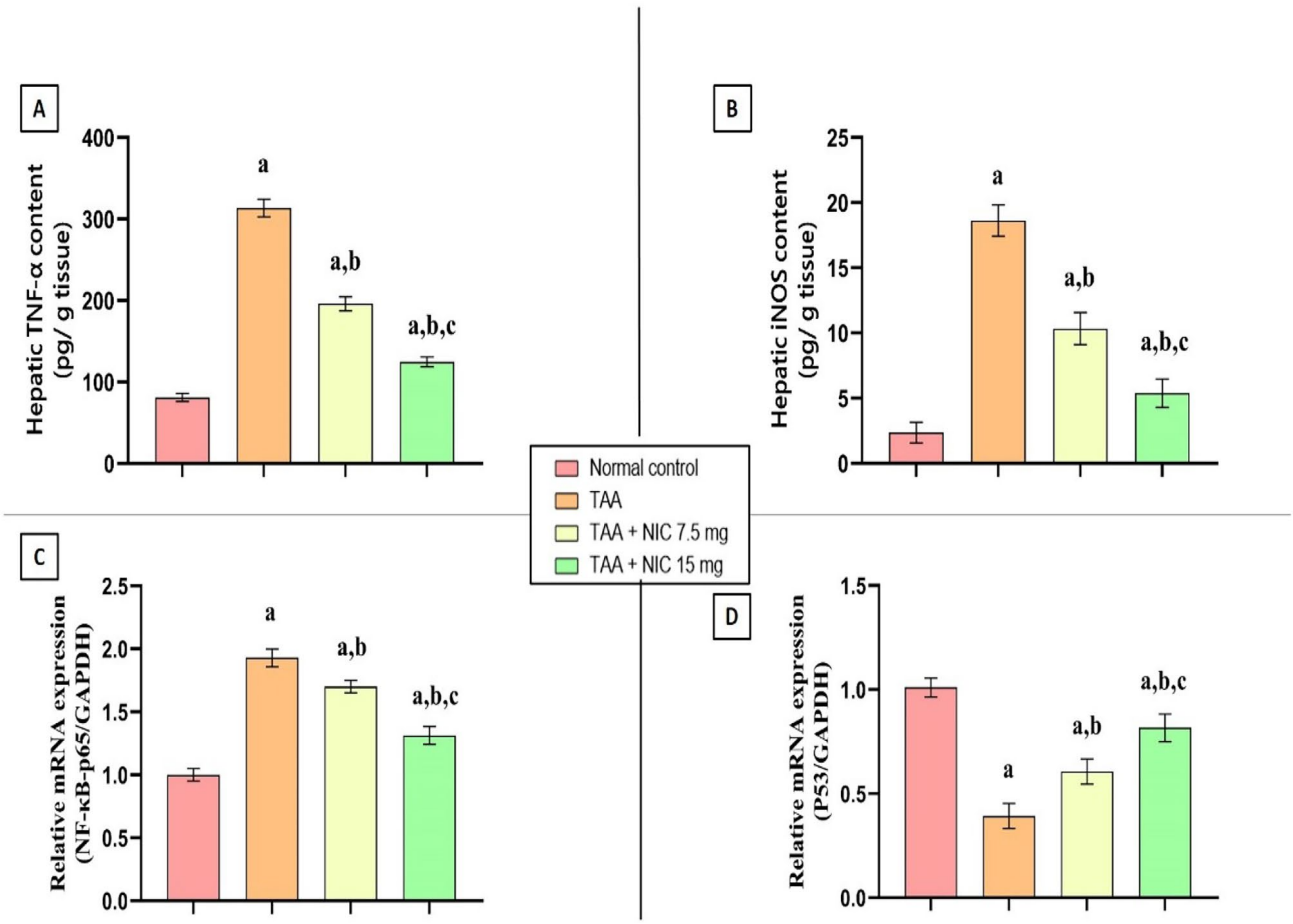
Effect of nicorandil (NIC) on hepatic inflammatory markers and gene expression of NF-κB-p65 and p53 in TAA-induced liver fibrosis in rats. This figure shows the effect of NIC on **(A)** hepatic TNF-α content, **(B)** iNOS content, **(C)** NF-κB-p65 mRNA expression, and **(D)** p53 mRNA gene expression in rats treated with TAA. Data are expressed as mean ± SEM (*n* = 6 per group). ^a^Significantly different from the normal control group (*p* < 0.05). ^b^Significantly different from the TAA group (*p* < 0.05). ^c^Significantly different from the TAA + NIC (7.5 mg/kg) group (*p* < 0.05).

As shown in Fig. 4, TAA administration led to a substantial increase in hepatic levels of TNF-α and iNOS, along with an upregulation of NF-κB-p65 gene expression. Conversely, P53 gene expression was significantly downregulated compared to the normal control group. Specifically, TAA exposure resulted in a 4.04-fold increase in TNF-α, a 6.67-fold rise in iNOS, and a 2.03-fold increase in NF-κB-p65 expression, while P53 expression was reduced by 50% compared to the normal control (Fig. 4A–D).

Treatment with NIC at a dose of 7.5 mg/kg led to a 33.25% reduction in TNF-α protein expression, a 41.42% reduction in iNOS protein expression, and a 30.24% decrease in NF-κB-p65 gene expression, while P53 gene expression increased by 50.04% compared to the TAA-induced liver fibrosis group. The higher NIC dose resulted in even more significant reductions in TNF-α and iNOS protein expression and NF-κB-p65 gene expression, with reductions of approximately 50%, 65%, and 50%, respectively, relative to the TAA-induced liver fibrosis group. This was also accompanied by a full restoration of P53 gene expression to levels comparable to the normal control group (Fig. 4A–D).

### Effect of nicorandil on the expression of COX-2 protein in hepatic tissue of rats with thioacetamide-induced liver fibrosis

**Fig. 5:**
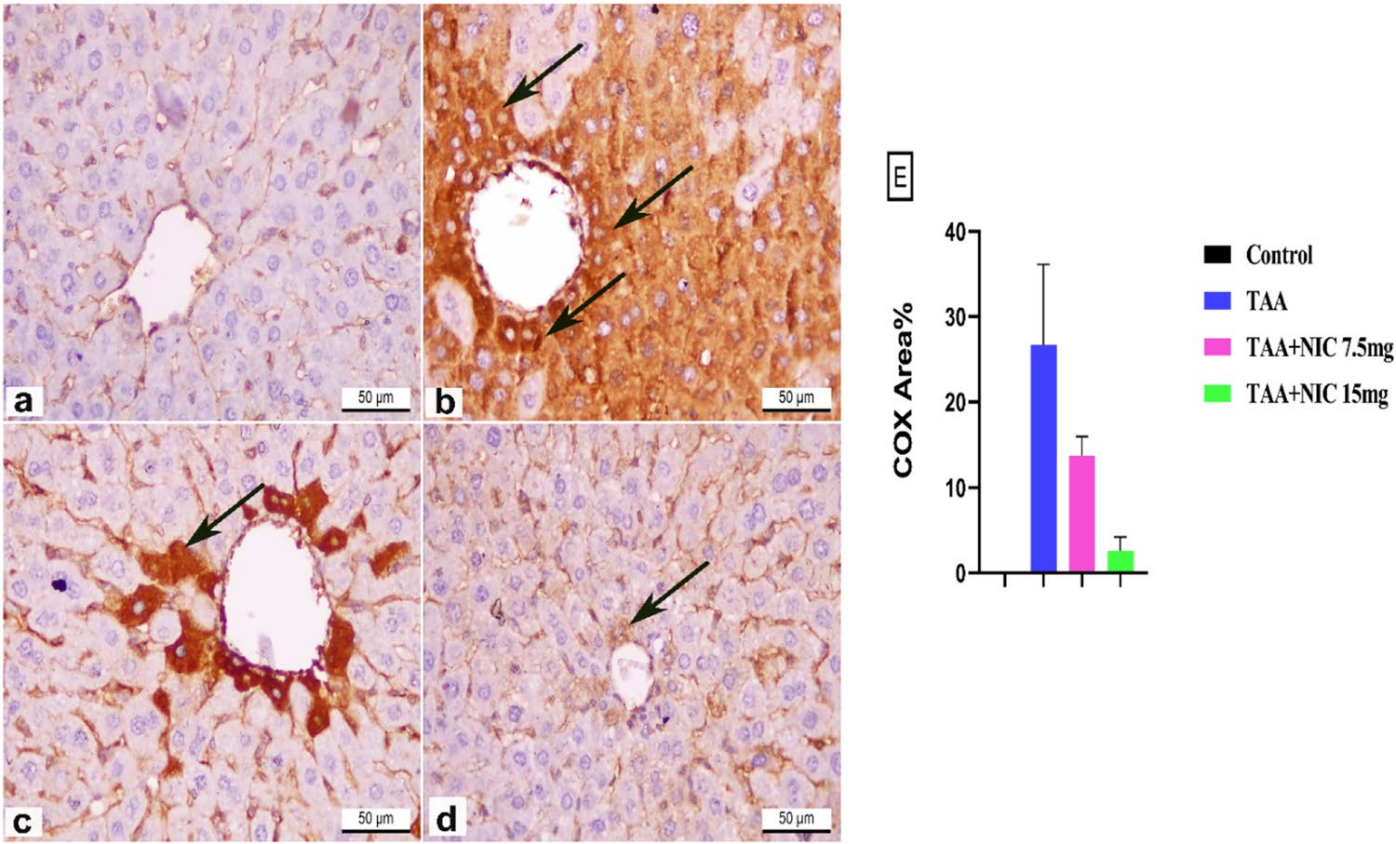
Effect of nicorandil (NIC) on hepatic expression of COX2 in rats with thioacetamide (TAA)-induced liver fibrosis showing COX2-stained hepatic sections of; **(a)** Normal control rats with negative immunoreactivity, **(b)** TAA treated rats with strong positive immunoexpression, **(c)** TAA + NIC 7.5 mg treated rats with moderate immunoexpression, **(d)** TAA + NIC 15 mg treated rats with mild immunoexpression, and **(e)** Bar chart representing the quantitative analysis of COX-2-positive stained area (%), clearly illustrating the differential expression levels among the groups. Data are expressed as mean ± SEM (*n* = 6 per group). ^a^Significantly different from the normal control group (*p* < 0.05). ^b^Significantly different from the TAA group (*p* < 0.05). ^c^Significantly different from the TAA + NIC (7.5 mg/kg) group (*p* < 0.05).

In our study, we observed that TAA administration led to a pronounced upregulation of COX-2 protein expression in hepatic tissues. This was evidenced by intense brown immunohistochemical staining predominantly localized around the central vein and in the hepatocyte cytoplasm (Fig. 5b). Quantitatively, the COX-2 positive area percentage increased significantly by 5.27-fold compared to the normal control group (Fig. 5a), which exhibited negative COX-2 immunoexpression (Fig. 5e).

Treatment with NIC at doses of 7.5 and 15 mg/kg effectively attenuated COX-2 expression in a dose-dependent manner (Fig. 5c, d). Rats treated with TAA and NIC at 7.5 mg/kg displayed moderate COX-2 staining in the hepatocyte cytoplasm, with a significant reduction of 51.20% compared to the TAA group. Moreover, the NIC 15 mg/kg treatment further reduced COX-2 expression to a mild level, with a 79.02% reduction compared to the TAA group, resulting in a COX-2 staining pattern nearly comparable to that of the normal control group (Fig. 5e).

### Effect of nicorandil on the pathological state of hepatic tissue of rats with thioacetamide-induced liver fibrosis

**Fig. 6:**
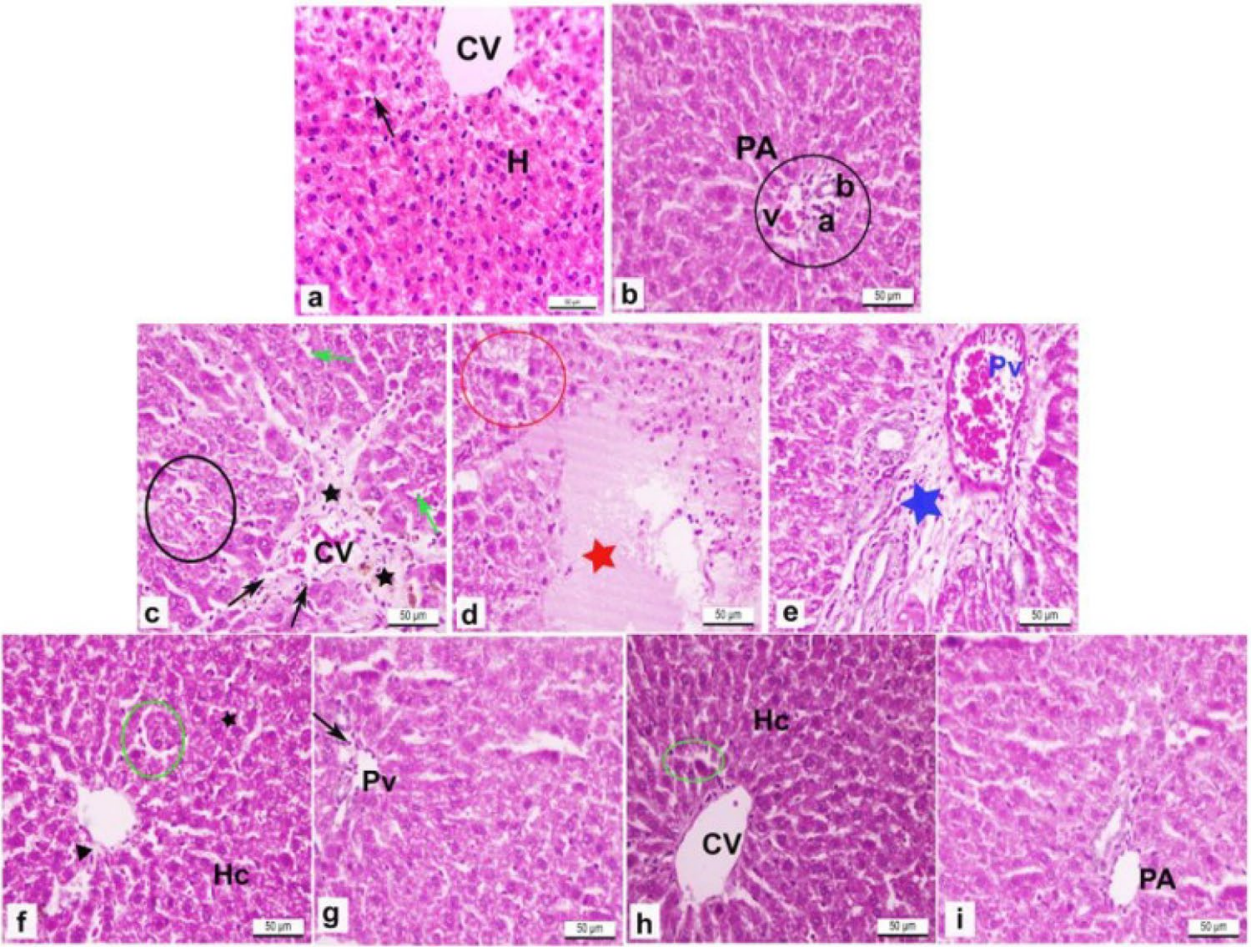
Effect of nicorandil (NIC) on hepatic histoarchitecture in rats with thioacetamide (TAA)-induced liver fibrosis using hematoxylin and eosin (H&E) staining. (**a**,**b**) Normal control liver sections showing intact hepatic cords, normal central vein (CV), and preserved sinusoidal structure. (**c**–**e**) TAA-treated rats displaying severe histopathological alterations, including disorganized hepatic cords, dilated sinusoids (black arrow), hepatocellular degeneration, and focal necrosis (red star), with marked alterations around the central (black circle) and portal veins (blue star). (**f**,**g**) TAA + NIC (7.5 mg/kg) group demonstrating partial histological improvement, with reduced sinusoidal dilation (green arrow), improved hepatic architecture, and fewer degenerative changes. (**h**,**i**) TAA + NIC (15 mg/kg) group showing marked recovery, with nearly normal hepatic cords, central vein (cv), and portal areas (PA), and minimal hepatocytes disarray (circle). Original magnification ×400.

Histological analysis of H&E-stained liver sections revealed distinct alterations across the experimental groups (Fig. 6). In the control group, hepatic tissue displayed normal architecture with hepatic cords radiating from the central vein. The hepatocytes were polygonal with large vesicular nuclei, separated by well-defined sinusoids lined by endothelial and Kupffer cells (black arrow) (Fig. 6a). The portal area exhibited a typical arrangement comprising the portal vein, hepatic artery, and bile duct (Fig. 6b).

In stark contrast, liver sections from TAA-treated rats demonstrated severe pathological changes, including disrupted hepatic cords, dilated sinusoids (black arrow) (Fig. 6c). Degeneration of hepatocytes, focal necrotic areas (red star) (Fig. 6d), and a dilated, congested portal vein branch accompanied by extensive portal fibrosis (blue star) were also prominent (Fig. 6e).

Meanwhile, Treatment of rats with 7.5 mg/kg of NIC partially alleviated these alterations. The hepatic architecture showed noticeable recovery with reduced fibrosis, less dilated sinusoids (green arrow), and almost normal hepatic cords, although some disorganized plates and degenerated hepatocytes were still evident (Fig. 6f). The portal area appeared nearly restored, with minimal collagen deposition (black arrow) around the portal vein branch (Fig. 6g).

Notably, administration of 15 mg/kg of NIC in TAA-treated rats resulted in substantial histological improvement. The hepatic parenchyma showed nearly normal cords, sinusoids (black arrow), and central veins, with only minor disorganization of hepatic plates. Similarly, the portal area was restored to near-normal conditions (Fig. 6h, i). These observations demonstrate the dose-dependent protective effects of NIC against TAA-induced hepatic damage. However, since our study assessed outcomes at a single timepoint (6 weeks), we cannot determine whether nicorandil primarily prevents the progression of fibrosis or facilitates reversal of established lesions. Thus, our findings should be interpreted as evidence of attenuation of fibrosis rather than definitive proof of reversal.

## Discussion

The current study demonstrates the protective potential of Nicorandil (NIC) in alleviating liver fibrosis induced by TAA in rats, with a particular focus on the modulation of the AMPK/SIRT-1/HIF-1α signaling axis. Our findings suggest that NIC modulates a multifactorial therapeutic effect that mitigates hepatic fibrogenesis, attenuates oxidative stress, and rebalances inflammatory signaling. These results position NIC as a promising pharmacological agent for the treatment of chronic liver diseases, potentially through its ability to restore cellular homeostasis and limit the progression of fibrosis.

TAA-induced liver fibrosis is a well-established model of chronic hepatic injury, characterized by ECM deposition, activation of hepatic stellate cells (HSCs), and disruption of normal liver architecture^25^. In the current study, TAA administration resulted in classical hallmarks of fibrosis: significant upregulation of TGF-β1 and COL1α1, marked collagen accumulation as shown by Masson’s trichrome staining, elevated hepatic hydroxyproline content, and severely altered histoarchitecture. Furthermore, liver function was compromised, as indicated by elevated serum ALT and AST levels. These findings are consistent with previous reports confirming TAA’s ability to induce chronic liver injury via sustained activation of hepatic stellate cells (HSCs), ECM accumulation, and deterioration of liver function^20,26^.

The first critical observation in our study is the clear anti-fibrotic effect of NIC. This response was supported by the downregulation of TGF-β1, a master fibrogenic cytokine, and the suppression of COL1α1 expression, both of which are key indicators of HSC activation. Histopathological findings corroborated these biochemical changes, demonstrating improved hepatic architecture and reduced fibrotic bridging in NIC-treated groups. Moreover, the liver function was markedly restored by NIC, as shown by the significant reduction in ALT and AST levels. These data collectively suggest that Nicorandil counters TAA-induced liver injury not only by structural modulation but also through functional recovery. Our results are in agreement with previous studies that reported the antifibrotic effect of NIC against various organ-induced fibrosis models^27–29^.

A pivotal finding in this study is the critical involvement of the AMPK/SIRT-1/HIF-1α signaling axis and its downstream effectors, particularly PGC-1α and PPAR-γ in mediating the hepatoprotective effects of NIC. This axis is central to hepatic adaptation under metabolic and oxidative stress, modulating antifibrotic and mitochondrial protective responses. AMP-activated protein kinase (AMPK), a master metabolic sensor, modulates protective mechanisms by promoting mitochondrial biogenesis, enhancing autophagy, and inhibiting pro-fibrotic pathways^30^. Under physiological conditions, AMPK activation is known to suppress hepatic stellate cell (HSC) activation and support mitochondrial integrity—both of which are crucial in mitigating fibrosis progression (An et al., 2024). Furthermore, AMPK positively regulates the expression of SIRT-1, a NAD⁺-dependent deacetylase that modulates transcription factors associated with oxidative stress, inflammation, and metabolic homeostasis^31^.

SIRT-1, in turn, deacetylates and activates peroxisome proliferator-activated receptor gamma coactivator-1 alpha (PGC-1α). PGC-1α is a master regulator of mitochondrial biogenesis and oxidative metabolism, and it forms a critical link between energy sensing and antioxidant defense mechanisms^32^. Furthermore, PGC-1α coactivates PPAR-γ, a nuclear receptor with well-documented anti-inflammatory and antifibrotic properties in hepatic tissue. Notably, both PGC-1α and PPAR-γ negatively regulate the activity of HIF-1α, either directly or via improved mitochondrial respiration and reduced ROS production, thereby limiting HIF-1α-driven fibrogenic and angiogenic signaling under hypoxic stress^33^. Consequently, dysregulation of the AMPK/SIRT-1/HIF-1α axis is increasingly recognized as a key contributor to chronic hepatic injury and fibrosis.

In our study, TAA intoxication significantly downregulated the hepatic expression of AMPK, SIRT-1, PGC-1α, and PPAR-γ, while upregulating HIF-1α, reflecting a shift toward mitochondrial dysfunction, impaired adaptive signaling, and fibrogenesis. Our findings suggest that nicorandil is associated with modulation of the AMPK/SIRT-1/HIF-1α signaling axis, which may contribute to its hepatoprotective effects. While the observed molecular changes are consistent with activation of this pathway, we cannot exclude the possibility that these effects are secondary to reductions in oxidative stress or inflammation. Thus, our data support a probable role of this signaling axis but do not provide definitive proof of direct AMPK activation. These molecular alterations were paralleled by histological and biochemical improvements, including reduced collagen deposition and improved liver architecture. Our findings are consistent with earlier reports demonstrating that AMPK activation inhibits TGF-β1-mediated profibrotic signaling, while SIRT-1 activation mitigates HIF-1α-driven angiogenesis and matrix remodeling^34,35^. These findings align with earlier studies demonstrating the ability of NIC to activate AMPK-dependent pathways, reduce hepatic oxidative injury, and inhibit fibrogenic gene expression in chemically induced liver injury models^11,28,36^.

Building on this mechanistic axis, the interplay between AMPK/SIRT-1/PGC-1α/PPAR-γ signaling and oxidative stress emerges as a central determinant in the progression and resolution of hepatic fibrosis. Oxidative stress is a well-established driver of liver fibrogenesis, contributing to mitochondrial dysfunction, hepatic stellate cell (HSC) activation, and excessive extracellular matrix deposition. In our model, TAA administration led to a significant elevation in hepatic malondialdehyde (MDA) and nitric oxide production (NOx) activity, alongside depletion of glutathione (GSH) and superoxide dismutase (SOD), indicating disrupted redox homeostasis and enhanced ROS production. These observations are consistent with previous findings showing that TAA induces lipid peroxidation and suppresses endogenous antioxidant defenses in the liver^20,37^.

Notably, NIC treatment in our study significantly restored hepatic antioxidant defenses, as evidenced by increased levels of GSH and SOD, along with marked reductions in oxidative stress markers such as MDA and NOx. These results align with growing evidence supporting Nicorandil’s antioxidant efficacy in experimental models of liver injury. For instance, Abdel-Sattar et al., 2020 demonstrated that NIC attenuated oxidative damage in a CCl4-induced liver fibrosis model by reducing MDA, enhancing GSH and SOD activities, and downregulating NOX expression—effects attributed to activation of the AMPK/SIRT-1 signaling pathway^27^. Similarly, Essam et al. (2025) reported that NIC alleviated oxidative stress and restored antioxidant capacity in a murine model of acetaminophen-induced hepatic encephalopathy, primarily via stimulation of the PI3K/Akt/eNOS axis^38^. These findings collectively suggest that NIC may exert its antioxidant and cytoprotective actions through multiple interconnected mechanisms, including both metabolic and redox-sensitive pathways.

Although prior studies have established that PGC-1α overexpression confers protection against hepatic injury by activating PPARα and PPARγ, thereby promoting the expression of key antioxidant enzymes such as SOD, CAT, and GPX^39^, no previous research has directly linked NIC to the modulation of this regulatory axis. This represents a major novel insight of our study, wherein we provide the first direct evidence that NIC treatment upregulates both PGC-1α and PPAR-γ expression, uncovering a previously uncharacterized mechanism of its hepatoprotective action. Moreover, the concurrent reversal of TAA-induced oxidative stress markers and activation of the AMPK/SIRT-1/PGC-1α/PPAR-γ axis in our model strongly supports the hypothesis that NIC exerts its protective effects, at least in part, through restoration of redox homeostasis and suppression of pro-oxidant signaling cascades.

Signal transducer and activator of transcription 3 (STAT-3) stands at the crossroads of inflammation and fibrogenesis, acting as a central regulator in the progression of chronic liver injury. In the current study, TAA administration markedly elevated hepatic STAT-3 expression, reflecting its pathological activation in response to oxidative and inflammatory stress. Persistent STAT-3 signaling is known to amplify hepatic inflammation by promoting the transcription of key pro-inflammatory mediators such as TNF-α, IL-6, IL-1β, and iNOS—molecules that further sustain hepatic injury and promote fibrotic remodeling through hepatic stellate cell activation^40,41^.

Treatment with NIC significantly attenuated STAT-3 expression, suggesting a key mechanism by which NIC disrupts this inflammatory amplification loop. This suppression of STAT-3 was accompanied by a marked downregulation of TNF-α, IL-1β, IL-6, and iNOS levels, reinforcing the centrality of STAT-3 inhibition in modulating the hepatic inflammatory landscape. Notably, previous studies have shown that NIC mitigates inflammation and fibrosis by inhibiting STAT-3 signaling in models of liver injury^18,27^. In parallel, NIC significantly upregulated IL-10, a potent anti-inflammatory cytokine known to counteract the effects of STAT-3–driven pro-inflammatory signaling and promote immune homeostasis. The increase in IL-10 is consistent with findings from other studies showing that NIC enhances the production of IL-10 to promote anti-inflammatory responses in hepatotoxicity models^42^. Moreover, NIC’s significant downregulation of COX-2 expression—a key mediator of prostaglandin-driven inflammation—further highlights NIC’s ability to suppress inflammatory responses at multiple molecular levels, which has also been observed in vascular and liver injury models^43^. These findings align with prior evidence indicating that STAT-3 inhibition can attenuate both inflammation and fibrogenic signaling in experimental liver fibrosis models^41,44^. Thus, by targeting STAT-3 as an upstream inflammatory driver, NIC modulates a broad anti-inflammatory response, restoring hepatic immunological balance and creating a molecular environment that favors tissue protection, regeneration, and resolution of fibrosis.

From a translational perspective, the effective 15 mg/kg dose in rats corresponds to a human equivalent dose (HED) of ~ 2.4 mg/kg based on body surface area conversion, which equals ~ 170 mg/day for a 70-kg adult^45,46^. Although higher than the currently approved clinical dose of nicorandil (up to 40 mg/day), this estimate suggests that the experimental dose lies within a potentially feasible range, warranting further pharmacokinetic and safety evaluations.

### Study limitation

This study has several important limitations that should be acknowledged. First, the mechanistic conclusions regarding AMPK activation are based on association and not direct causation, as pharmacological inhibition or genetic silencing experiments were not performed. Second, only a single experimental timepoint (6 weeks) was assessed, which prevents distinguishing between preventive versus reversal effects of nicorandil on fibrosis. Third, histopathological assessment of H&E sections was descriptive and not based on validated semi-quantitative scoring systems, although morphometric quantification of collagen (Masson’s trichrome) and COX-2 immunoexpression provided objective measures. Fourth, human equivalent dose (HED) calculations and long-term safety margins were not experimentally addressed, and translation requires cautious evaluation. Finally, although the TAA protocol produced biochemical, molecular, and histological hallmarks of fibrosis, additional validation using standardized scoring systems would further strengthen the distinction between fibrosis and acute hepatotoxicity. These limitations should be considered when interpreting our findings, and future studies are warranted to address them through multi-timepoint designs, pathway inhibition approaches, and standardized fibrosis scoring systems.

## Conclusion

Our study demonstrates the significant hepatoprotective potential of NIC in TAA-induced liver fibrosis, with evidence of a dose-dependent response—where the 15 mg dose provided superior outcomes. NIC effectively restored liver function, mitigated oxidative stress, reduced inflammation, and suppressed fibrotic progression. These effects were mediated, in large part, through modulation of the AMPK/SIRT-1/HIF-1α signaling axis, which is central to cellular regeneration and inhibition of fibrogenesis. By targeting multiple pathological mechanisms simultaneously—oxidative damage, inflammatory signaling, and fibrotic remodeling—NIC shows potential as a therapeutic approach for liver fibrosis. However, these findings are limited by the use of a single animal species, an acute liver fibrosis model, and the absence of comprehensive toxicity assessment. Therefore, further studies are needed to confirm these results in chronic models, assess safety and dosing, and evaluate translational relevance in human populations.

## Supplementary Information

Below is the link to the electronic supplementary material.

Supplementary Material 1

## Data Availability

The data that support the findings of this study are available from the corresponding author upon reasonable request.
