# Supplementary figures and images for: Nicorandil attenuates thioacetamide induced liver fibrosis via AMPK, SIRT1 and HIF1α mediated cellular energy homeostasis
Source: Sci Rep. 2025 Dec 9;15:43477. doi: 10.1038/s41598-025-28309-7 (PMC12695948; doi:10.1038/s41598-025-28309-7)

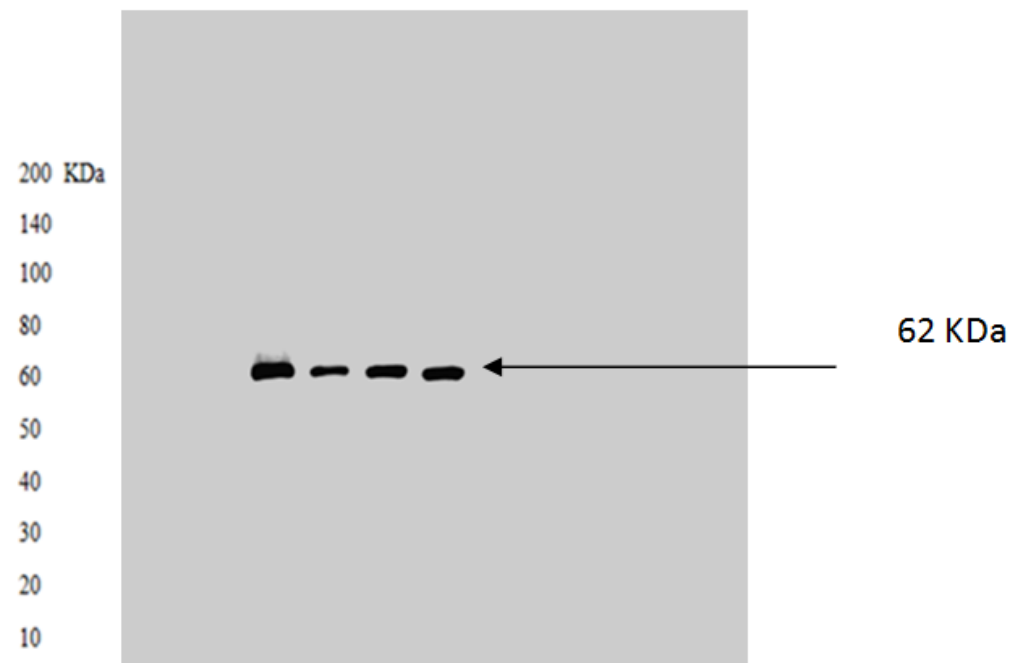

**AMPK**

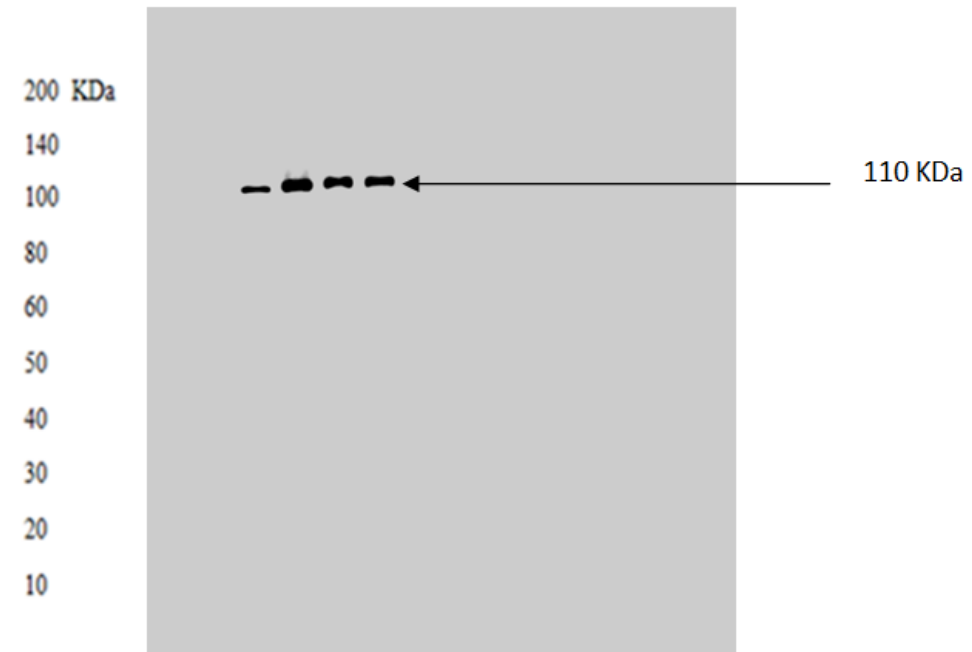

**HIF-1 $\alpha$**

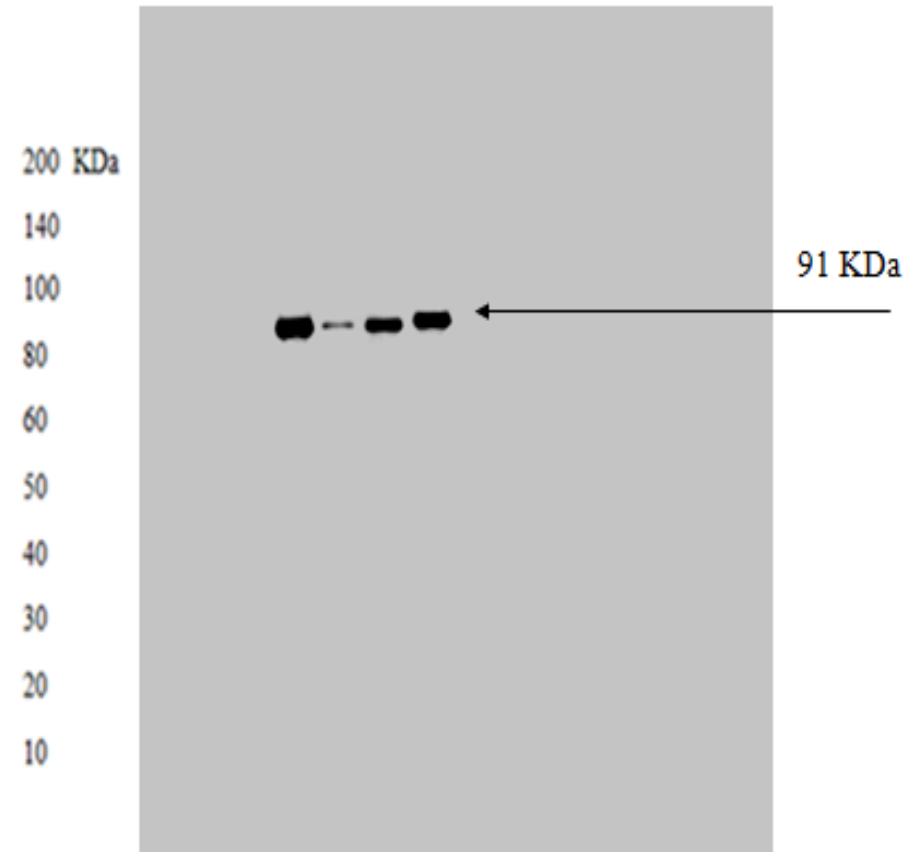

**PGC-1 $\alpha$**

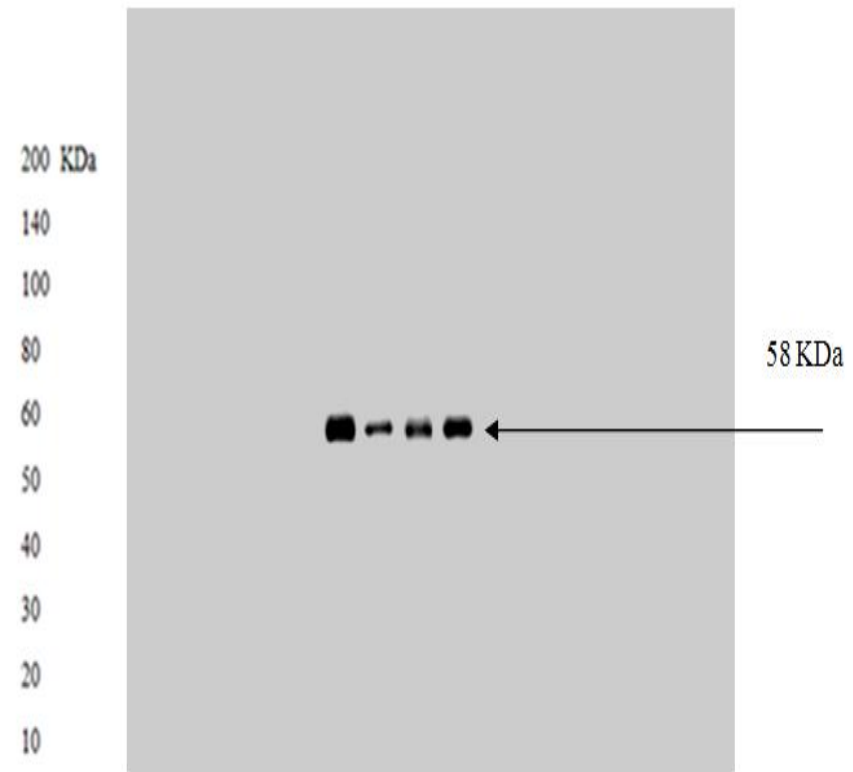

**PPAR-gamma**

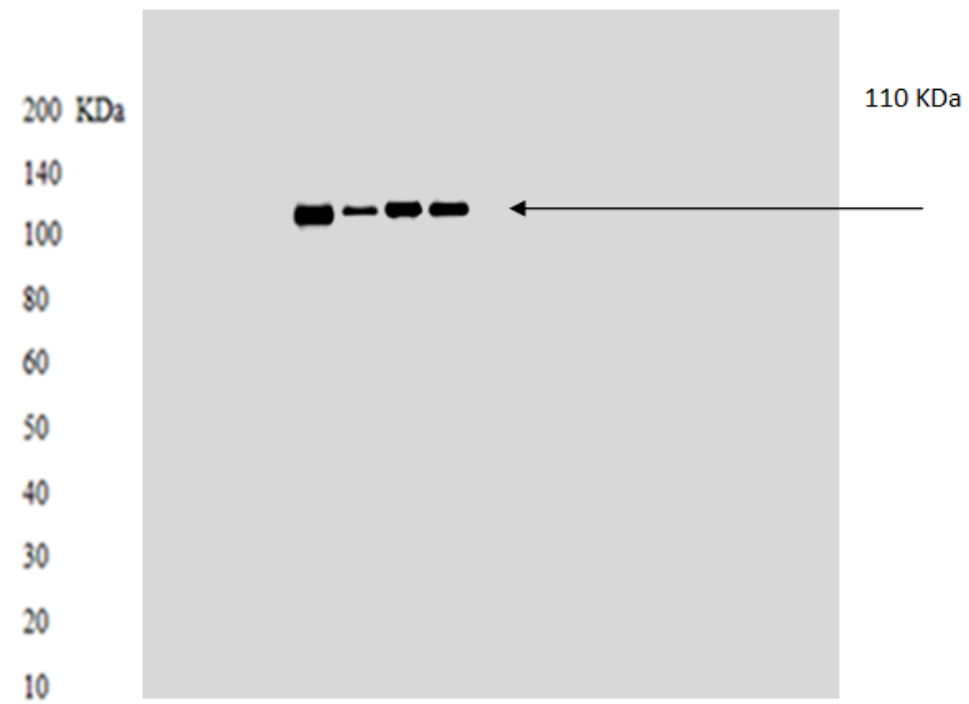

**SIRT-1**

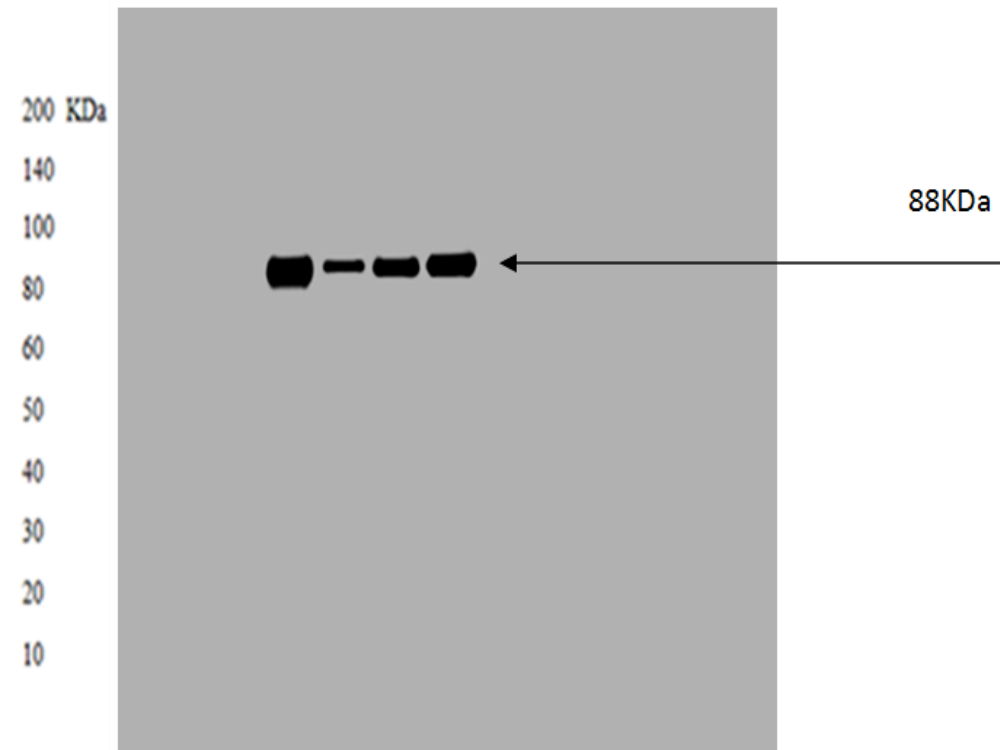

**STAT3**

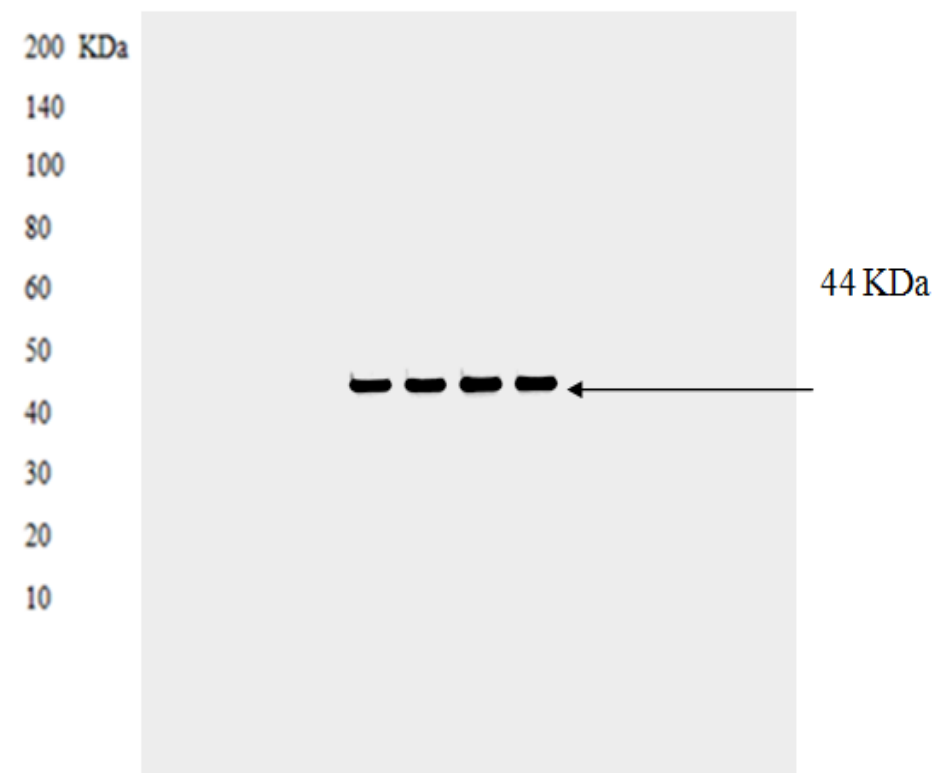

$\beta$ -actin

Supplement: Supplementary file 1 — Supplementary Material 1 [file 41598_2025_28309_MOESM1_ESM.pdf]
